# Schoolgirls’ health agency: silence, upset and cooperation in a psycho-educational assemblage

**DOI:** 10.1080/17482631.2018.1564518

**Published:** 2019-01-20

**Authors:** Anette Wickström

**Affiliations:** Department of Thematic Studies, Child Studies, Linköping University, Linköping, Sweden

**Keywords:** Schoolgirls, health agency, psychological health, psycho-education, assemblage ethnography

## Abstract

**Purpose:** Since the millennium, manual-based preventive health programmes, drawing on psychological models of behaviour management, have dominated psycho-educational practices in school. The aim of this article is to study the health agency of 13-year-old schoolgirls participating in a programme for improving schoolchildren’s psychological health in Sweden.

**Method:** Drawing on Deleuze and Guattari’s theories of assemblages, the interaction between schoolchildren, teachers, the manual and psycho-educational techniques is scrutinized. The methodology of assemblage ethnography is used in the analysis of video observations of 13 course meetings.

**Results:** Three salient attitudes in relation to the possibilities built up for the schoolgirls are identified—*silence*, *upset* and *cooperation*. The girls’ acts and stories question the psycho-centric, individualized and gender-normative approach used in psycho-educational programmes and make visible the relational and contextual aspects of schoolchildren’s psychological health.

**Conclusion:** Children depend on multiple factors for their agency; the institutional networks they are involved in both allow and restrict their actions. The study demonstrates that focusing on children as health actors, in the sense that agency develops in the assemblages children take part in, can complement the knowledge base and question the predominant framing of psychological health.

## Introduction

I am sitting in a classroom together with a teacher and seven 13-year-old girls participating in a psycho-educational programme in school. The teacher and the schoolgirls are sitting on chairs in a circle in the middle of the room. I have put up two cameras on tripods and taken a seat at a desk by the windows. The teacher explains to the girls that she is going to teach them a method for preventing negative thoughts and thinking more positively about themselves. They are going to do an exercise in which they paste a piece of paper on their backs on which the other participants will write two positive points that match the person. The teacher puts on music and the girls paste the paper on their backs, walk round slowly and write some words on each other’s backs. After a while the girls stop, remove the papers from their backs and start to read what is written there to themselves.

Teacher: Is there anybody who would like to read what is written to the others? (Everybody is quiet. Josefin puts her paper on the floor and looks at her cellphone.)

Sara: No.

Josefin: Don’t want to.

Teacher: You don’t want to? Is there anybody else who does not want to, or is there anybody who would like to? (Everybody is quiet.)

This excerpt is taken from a research project on a manual-based psycho-educational programme in a Swedish school; it demonstrates a contradictory attitude among the participating 13-year-old girls. They take part in the course meetings but are reluctant to share their thoughts as expected in many of the course exercises. Their attitudes and agency are in focus for this article, as is the psycho-educational programme, which is used in many Swedish schools. The programme is called DISA (Swedish acronym for Activate Your Inner Strength) (Clarke & Lewinsohn, 1995; Lindberg, Svensson, Treutiger, Koertge, & Thomas, 1995) and it aims to help schoolchildren develop positive thinking skills. In this article I study the educational programme with a specific focus on how the participants both challenge and adhere to it. But first, a brief historical overview of such programmes.

At the end of the 1990s a shift occurred in the political debate in Sweden on children’s vulnerability. Previously, the worries in discussions on children’s needs and development had principally concerned children who were the victims of problematic situations and home conditions (Littmarck, 2017). The political debate shifted towards children who risked developing psychological ill health. Alongside this shift, public health initiatives were aimed at children in general (Kvist Lindholm, 2015). No single child living under harsh conditions would any longer be stigmatized and singled out as socially disadvantaged. Instead, public health politics promoted universal initiatives directed towards whole populations of children in order to prevent psychological ill health. In this respect, schools became the “natural” sites for health promotion (World Health Organization, 2015; Wright, 2015). To reduce the risk of depressive disorders in children and young people, manual-based psycho-educational programmes were implemented (Kvist Lindholm, 2015; Merry, McDowell, Hetrick, Bir, & Muller, 2009). Programmes that draw on psychological models of behaviour management have dominated the psycho-educational practices in schools (e.g., Kvist Lindholm, 2015; Watson, Emery, Bayliss, Boushel, & McInnes, 2012). Schoolchildren are encouraged to observe themselves, register their thoughts and emotions and try to take control over them; that is, to learn techniques to improve their psychological health. Instead of relating children’s vulnerability to socio-economic factors, the children are positioned as being the source of the problem. Consequently, children have the responsibility to work on themselves as a preventive measure and to avoid the risk of suffering from psychological ill health in the future.

Several educational scholars refer to this phenomenon as a period of “programme invasion” of Swedish schools (e.g., Bartholdsson & Hultin, 2015). It is suggested that the establishment of the programmes internationally marks a “therapeutic turn” in education (e.g., Coppock, 2011; Wright, 2015). Issues concerning children’s health and well-being have been dealt with on contract in many schools, which means that the activities bear the stamp of values, norms and methods defined by actors, and used in practices, outside the education sector (Watson et al., 2012; Wickström, 2012). In this respect, however, research has demonstrated that the programmes are attractive in as much as they show that principals and teachers wish to show that they care for the schoolchildren’s psychological health, that they strive for academic standards and that it is difficult for them to question the programmes (Watson et al., 2012). Teachers become positioned foremost as instruments through which manual-based instructions can be realized and a healthy development of the population promoted (Wickström, 2012). By directing teachers’ attention towards remedying assumed deficits in social and emotional skills in students, the programmes neglect the broader social, relational and cultural contexts of schools (e.g., Bartholdsson, Gustafsson-Lundberg, & Hultin, 2014; Wickström, 2013). However, long-term work by the school staff in close collaboration with the schoolchildren is demonstrated to be more effective than pre-produced programmes (Söderström, 2013).

In the present article, I draw on an ethnographic study of a DISA programme carried through in school, in order to situate a study of agency within an institutional network. The study was part of a collaboration project carried out between a research group at Child Studies at Linköping University and a municipality in central Sweden to evaluate public health interventions for children and young people. My role was to study the realization of DISA (see Wickström, 2012, 2013). The aim of the programme is to improve teenage girls’ well-being and reduce the risk of them developing psychological ill health by teaching them positive thinking skills (Clarke & Lewinsohn, 1995). The reason why girls are the target in Sweden is that in a survey from 2009, girls reported more psychological health problems than boys (Folkhälsomyndigheten, 2011). DISA is derived from an American programme for depressed people and has been adjusted to suit Swedish conditions and reformulated as a preventive activity. The manual springs from cognitive behavioural therapy (CBT) and the theory that depressed people have automatized negative notions of themselves and their surroundings, and that these notions must be reformulated (Beck, 1979). Consequently, the exercises consist mainly of identifying negative thoughts and trying to change them. A cure for depressed people is used as a preventive intervention for healthy schoolchildren.

The introductory episode demonstrates that the girls are interconnected with a number of relations. They take part in the DISA practice and the techniques used in the exercises. Their bodies are used for printing positive words in an exercise to change negative attitudes. The girls engage with, and are composed by, each other, the teacher, the room and the manual. In order to investigate the girls’ agency and the role of the psycho-educational programme, I use a socio-material approach (e.g., Lee, 2001; Prout, 2000). The socio-material approach highlights that actors can be of many different kinds: humans as schoolchildren and teachers but also non-humans such as manuals and educational techniques. Moreover, in order to emphasize children’s creative capacities to shape the institutional activities they take part in, I introduce the concept of children as health actors. The aim of the article is to investigate how schoolgirls act when they take part in the intervention, and to get an understanding of their experiences of psychological health. In addition, the aim is to reflect on how psycho-educational practices, intended to improve children’s psychological health, open up possibilities and set limits for children’s agency.

## Literature review

### Sociology of childhood: agency and assemblages

Within the sociology of children and childhood, researchers have argued for an actor-oriented perspective of children’s and young people’s health (e.g., Brady, Lowe, & Olin Lauritzen, 2015; Halldén, 2003; James & Hockey, 2007; Mayall, 1998). The aim has been to direct attention to children and young people themselves, their actions and how they experience and reason about their health, thus including children’s and young people’s own perspectives. This is of particular relevance to psychological health, which constitutes the very foundation for children’s and young people’s subjectivity and identity making. Children’s and young people’s experiences of feeling psychologically well or not need to be heard and collected as basic information in scientific studies (e.g., Armstrong, 2000; Liegghio, Nelson, & Evans, 2010). Attending to children and young people as health subjects implies first of all a holistic view of psychological health (cf. Fattore, Mason, & Watson, 2012). It means that health is defined as closely tied to the individual’s possibilities and conditions in a wider social and cultural context (e.g., James & Hockey, 2007; Landstedt, Asplund, & Gillander Gådin, 2009). This holds true very much for psychological health, in the sense that expressions of psychological problems or ill health are related to the child or the young person and his or her relational network and sociocultural context.

The question of children’s agency in relation to their body and health has raised interest in how children are active and engaged when they participate in daily life, but also interest in the factors they depend on for their possibilities to act. Studies of children’s health agency, however, have mostly concerned the identities of sick children (e.g., James & Hockey, 2007) or young people engaged in, for example, cosmetic surgery and fitness training (e.g., Coffey, 2016). This article addresses instead the way children and young people act and contribute to their own well-being when targeted by institutionalized measures intended to improve their psychological health. In the following, I draw on philosophical theories used by scholars in the field of children and childhood sociology. The basic argument is that a child does not exercise agency independent of its surroundings, but that agency is produced in socio-material practices and as part of power relationships (e.g., Lee, 2001). Children are actors in a social context in which they interrelate with peers, technology, social norms, body ideals and the health market, for example (cf. Coffey, 2016; Lee, 2001; Prout, 2005). Children may act as agents of change, but they are dependent on people and materiality for their ability to do so. In order to include socio-material aspects in the analysis of children’s agency, researchers need to attend to the networks in which children are deeply involved (Lee, 2001; Prout, 2005).

Schoolchildren participating in psycho-educational practices have relations to physical, social, cultural, political and ideological contexts. An interest in understanding what the relations do brings us to Deleuze and Guattari’s (1987) concept of *assemblage*. The approach involves shifting the focus from the dichotomous understanding of structure versus agency to interest in a body’s relationship with the social world and with numerous forces. A reconstructed theory of assemblages has been introduced as a way to “get a sense of the irreducible social complexity characterizing the contemporary world” (DeLanda, 2006, p. 6). Social assemblages can be interpersonal networks but also institutional organizations consisting of components such as people, food, buildings, rules, tools and machines. The components play variable roles and by exercising its capacity a component may alter the assemblage or be detached from it (DeLanda, 2006). An assemblage is formed through engagement, be it a school’s realization of a psycho-educational programme or schoolgirls’ decision to participate, and it is the outcome of a body’s relations and engagements (cf. Coffey, 2016, p. 29). The assemblage theory is meant to open up possibilities for rethinking the body and the social beyond dualistic frameworks (Coffey, 2016, p. 31). Schoolgirls participating in a psycho-educational programme connect to a variety of forces such as peers, teachers, gender norms, systems of education and health discourses (Wickström, 2013). Their engagement is productive in that they shape what will happen next—action, change, stasis (Coffey, 2016, p. 31). Assemblages of relations limit what a body can do, but they can still be the basis for resistance (Fox, 2012, p. 154). Children’s creativity and interests interact with the relations that affect a child, relations that at the same time open up possibilities and set the limits (see Coffey, 2016; Lee, 2001; Prout, 2005). Health interventions in school implement medical health discourses and health identities such as “positive thinker” or “girls’ problem” which limit what schoolgirls may do (Wickström, 2013). However, the intervention assemblage may also be affected, in that it provides a context that supports the teacher and helps the participants to resist and expand their possibilities (cf. Fox, 2012, p. 155). Conceptualized in this way, agency is variable and an effect of the connections between actors, educational techniques and spatial arrangements.

### Health actors

A decentralized concept of agency opens the door for empirical studies and explorations of when and how children exercise agency (Bollig & Kelle, 2016). It also raises questions about the dynamic character of assemblages: how they are formed, what elements they consist of and how “doings” may alter the assemblages (Fox, 2012, p. 67). Schoolchildren’s actions, a remade course manual, change of teacher or change of rooms are examples of “doings” that may change the assemblage. Theorizing these processes makes it relevant to study how agency is built for and used by children, in specific practices. Useful starting points for this purpose are the concepts of *territorialization/deterritorialization* and *coding/decoding* (DeLanda, 2006). Processes of territorialization and deterritorialization account for the variable role the assemblage plays. The capacities of the assemblage’s different elements can either stabilize the identity of the assemblage (by increasing its homogeneity) or destabilize it (by increasing its heterogeneity) and “forcing it to change or even transforming it into a different assemblage” (DeLanda, 2006, p. 12). Processes of coding/decoding consolidate the effects of territorialization/deterritorialization. Formal rules governing an assemblage make the social encounter coded, while, for example, participants getting room to express themselves may weaken the rules and start a process of decoding (cf. DeLanda, 2006, p. 16). I will use these concepts when analysing what is happening in the educational assemblage in focus in this article. Assemblage theory widens the focus on agency and takes an interest not only in human action, but also in the agentic role of technology, buildings, rooms, tools, etcetera. “Language plays an important part but not a constitutive role”, argues DeLanda (2006, p. 3). This widened perspective has directed my interest to what the manual, the room and the exercises do. Nevertheless, the schoolgirls’ actions and verbal comments receive considerable attention in the analysis.

Interdisciplinary scholars studying children and childhood have long directed their interest towards the ways children and young people negotiate activities they take part in (e.g., James, 2007). Schoolchildren, for example, are accustomed to participate in activities in school. Most of them do not offer resistance or refuse to take part, but that does not prevent them from challenging and questioning some parts. Children always influence the activities, and to some extent change them when they participate (e.g., Viruru, 2007). In order to analyse schoolchildren’s influence on activities, it is important not only to study resistance, but also to try to see what is at the bottom of their polite or indifferent behaviour, how they hesitate about or avoid some parts of the activities and how they try to change or introduce alternative activities (cf. Viruru, 2007). In her classic study, Mayall (1998) suggests that empirical work needs to present children’s own experiences and develop the idea of children as *health care actors*, seeing how they take charge of their bodies, without losing sight of “the social and political issues central to childhood experience” (p. 283). In introducing the concept of *health actors*, I aim to expand the focus on children as service users or receivers of medical interventions to include children’s and young people’s experiences and negotiations of the multiple efforts by authorities and private entrepreneurs to prevent psychological ill health (cf. Brady et al., 2015). Seeing children and young people as health actors opens the door to alternative interpretations of psychological health and helps broaden our knowledge about how to deal with children’s psychological problems. In the present article I investigate the schoolgirls’ negotiations of a psycho-educational programme to see what they value and find fundamental to their psychological health. Their acts of interest, cooperation, opposition and elaboration of the course elements work as narratives of their experiences of psychological health. The practices also demonstrate how their health agency is produced in the assemblage of the educational programme.

### The agentic role of psycho-educational programmes

Interdisciplinary childhood scholars have drawn attention to the implicit normative and regulative dimension of psycho-educational techniques. One main issue raised is that the psycho-educational programmes constitute a categorization of emotions and experiences in terms of psychiatric symptoms (Kvist Lindholm, 2015). The way emotions and experiences are categorized influences how children and young people view themselves and how they give meaning to psychological health. The philosopher Ian Hacking (2004) has taken an interest in understanding what happens when diseases and their symptoms are defined. He uses the concept *making up people* to investigate how people are altered by being classified. The classifications of psychological health in psycho-education are not neutral. They work as assemblages that act on children and young people, who start to view themselves in relation to the classifications. Hacking (1995) demonstrates that when people change, they cause the classifications to change in turn. The concept of *looping effect* refers to the process of transformation initiated when psychiatric concepts such as depressive symptoms are given new meanings when people use them (Hacking, 1995). Morally loaded categories, such as the categories of psychological health problems used in the psycho-educational programmes, entail a strong *interactive process* (Hacking, 1995, p. 370). What Hacking (1999) finds important to understand is what knowledge about people does to people. In the case of psycho-education, it is crucial to see how the educational assemblage allow and restrict the schoolchildren who take part in them, and likewise how the schoolchildren reproduce, transform or alter the assemblage.

## Assemblage ethnography

Ethnographic methods have held an exceptional position within the sociology of children and childhood, and been valued as well suited to studying children’s and young people’s everyday lives and perspective, not least when it comes to psychological health (cf. Kostenius & Öhrling, 2006; Liegghio et al., 2010). The specific methodology called assemblage ethnography “aims to reach across government and policy networks; institutional and professional forms, practices and subjectivities; and the civic and social practice of people engaged in their everyday lives” (Youdell & McGimpsey, 2015, p. 120). Assemblage ethnography requires creative uses of methods and attention not only to visible actors, but also to other components that form an assemblage. In the project I draw from in this article, participant and video observations were used to study the different components in action when the educational programme was realized. In 2011, I travelled to a 9-year compulsory school in Sweden to present my study to all 13-year-old girls in the school. The two collaborating teachers had planned two DISA courses with the aim to let me do research in one of them. During the information meeting, one of the schoolteachers invited the girls to join a DISA course. I told them about the research project, the observations and the ethical questions of informed consent, confidentiality and voluntariness. Seven girls chose to be part of the research project. The course ran from January to May and included 13 classes. I used 2 cameras on tripods to video-record all the classes and I observed 11 of the classes and wrote field notes on what was going on. A graduated psychology student transcribed the videos verbatim.

When reading the transcriptions, I paid specific attention to the girls’ engagements, negotiations and contestations, and used actor-oriented interpretations to understand what was going on and what it meant to the participants (see Geertz, 1973, p. 14). In the first stage I coded every episode where the participants interacted with each other, the teacher and the exercises. This coding resulted in the identification of two processes: resistance and transformation. The girls resisted specific parts of the course and in collaboration with the teacher they introduced other activities than the ones indicated in the manual. During the analysis process I discussed the analysis in the research group. I compared my findings with the results from the analysis of individual and group interviews with 37 schoolgirls taking part in DISA (Kvist Lindholm, 2015; Kvist Lindholm & Zetterqvist Nelson, 2014). In second-degree interpretations (Fangen, 2005, p. 228) I brought in relevant contexts and theories to explore the processes. In this article I especially focus on the participating girls’ agency as produced in the assemblage of the psycho-educational programme. Using tools from assemblage ethnography, I investigate the productive relations between different assemblage components, how the girls act in relation to the course activities and how the school, the teacher and the DISA manual build agency for them. I illustrate their actions by looking at three salient attitudes in relation to the possibilities built for them: *silence*, *upset* and *cooperation*.

## Ethical considerations

A psycho-educational course in school might result in participants sharing specific concerns and emotions. To prepare for all contingencies I planned the observations together with my colleagues at Linköping University and with the teachers who were involved in the course. We discussed how to help facilitate support for the participants if they raised issues that needed to be addressed outside the research encounter. When it comes to the question of informed consent, I considered it not only as guaranteed before the girls were involved in the project, but as an ongoing research practice (Harcourt & Quennerstedt, 2014). I paid attention to the girls’ motivation during the course and opened up the possibility to not participate in the research. One of the girls chose to leave the course after some time.

While the study gave rise to ethical concerns addressed above, it also provided a possibility in which schoolchildren’s actions and views could be used to critically examine health interventions in school. One year after the course, I returned to the school to present my initial analysis to the participants. Five of the seven girls attended the meeting. I told them about my interpretation of what parts of the course they had resisted and what they had tried to achieve instead (Wickström, 2013). The girls expressed appreciation of getting the chance to talk about their perspectives. They seemed to be proud that their acts of protest and resistance were taken seriously—being seen as a source of knowledge rather than an obstacle to be overcome.

## Results

### Silence and reluctance

The form of the educational assemblage—the manual, the DISA trained teacher, the small group of schoolchildren and the well-defined spatial and temporal boundaries—constituted a process of territorialization (cf. DeLanda, 2006, p. 13). Material, professional and student roles and the room were involved in setting the boundaries for and the identity of the assemblage. The four first classes in DISA are supposed to present the main idea: that young girls have negative thoughts about themselves and their surroundings, thoughts that need to be transformed into positive ones (cf. Beck, 1979). These basic assumptions consolidate the effects of the territorialization in that they spell out the rules and code or strengthen the assemblage (cf. DeLanda, 2006, p. 16). By doing various exercises the participants are supposed to bring up examples of negative thoughts and discuss them in front of the group. The girls demonstrated in both words and deeds that they were not willing to work with their thoughts in accordance with the outline of the course. They sat in silence for long periods of time, refusing to answer the teacher’s questions. In problematizing the concept of voice in childhood studies, Spyros Spyrou (2015) emphasized that silence is an integral part of voice. Silences are pregnant with meaning and researchers need to pay attention to what is left out (Spyrou, 2016, p. 113). He attends to the unspoken and investigates the meaning of silence in order to escape the narrow focus on children’s voice as utterances. During my observations, the girls’ silences were the first thing that struck me. The girls’ silences literally “shouted” at me and I could not refrain from considering them.

At the fourth class the teacher renewed her efforts to get the girls to talk about their negative thoughts. This went on for 10 minutes. After that long time, the teacher tried to entice them into giving any examples by modifying the word negative and changing it into “something the others do not know” or “a cosy incident”:

Teacher: You will have the great honour to tell the others in the group **something, something** they do not know about you … Is there anything they perhaps do not know? … (everybody is quiet) I will also tell you a secret … that nobody else knows (everybody is quiet)/—/It could be a cosy incident, it could be … Who wants to start? (everybody is quiet) Do you want to start, Hilma?

Hilma (answers quickly): I have none.

Teacher: Have you got no secret, nothing?/—/You then, Sara?

Sara: Many, but I’m not allowed to tell them.

Teacher: No (laughs) Right … Do you have a secret (looks at Josefin) that you wish to tell?

Josefin: I’ve got one, but I’m not going to tell it (gripping her file).

Teacher: No, it can be like that … Have you got something, Mari?

Mari: No.

Josefin: It’s a bit private.

This excerpt demonstrates that a looping effect (Hacking, 1995) started when the teacher explained the exercise and tried to involve the participants. Since the teacher had met silence in four lessons, she developed the exercise. She turned the concept of negative thoughts into a secret or something cosy. The girls spoke up, but they still refused to answer the question. The girls explained their unwillingness in different ways, such as having nothing to tell or that the experience they had was private. None of them gave any example of negative thoughts during the 13 course meetings. The girls’ capacity to be silent, their expressions of ignorance and the teachers renewed efforts to get them to talk deterritorialized the educational assemblage in that it destabilized it (cf. DeLanda, 2006, p. 12).

The girls’ reluctance to tell any negative thoughts could, of course, be read simply as secretiveness or anxiety about exposing oneself. This reading, however, is contradicted when considering activities in which the girls were given room to decide on the topic for a conversation. On these occasions they talked about their personal problems and difficulties without hesitation, alongside stories about their interests and competencies. Another reading of their silence is that they did not fully understand the question or the task they were assigned, namely to tell negative thoughts and then turn them into positive ones. In practice, they were asked to *come up with thoughts they should not have* and then try to change them—a somewhat unapproachable process for them. The girls kept on being quiet for long periods. The ready-made problem focus in the DISA manual establishes a picture of individual thoughts being the problem. By constantly being silent and reluctant in relation to exercises where the participants are supposed to work on their thoughts, the girls seemed to reject the idea that locates psychological problems within the individual. The girls had other suggestions for what their problems were, as was demonstrated when they upset the course.

### Upset and make meaningful

The second aspect of the girls’ practices was that they used the time and the place that the programme offered (in the form of regularly recurrent classes) and to an extent that the teacher allowed for seeing each other and to talk about their interests and capacities. In that way they upset the course and made it meaningful. Instead of turning the focus inwards on themselves and noticing deficiencies, they turned their interest on each other. They directed the focus on the aspects that constitute the basis of their psychological health. The girls were busy interacting, demonstrating the importance of belonging and experiencing feelings of solidarity. Together with the teacher, who was flexible and tried to promote a constructive atmosphere, they made room for coffee breaks and extended the time for the few exercises they appreciated, such as “sharing”. In this exercise, the participants were asked to bring something they liked and share it with the others. In the group I studied, they shared cell phone pictures. However, the girls upset the exercise, which was supposed to be a short concluding exercise, as seen in the following excerpt:

Josefin: I have lots of nice pictures here.

Teacher: You have? It would be very nice if you showed us one later.

Josefin: Nice spring pictures.

Teacher: Listen, Josefin, I would like you to show them later, before we finish.

Josefin: There are many of them (smiles).

Teacher: Yes, but you can choose three.

Elin: I would also like to show some pictures.

Teacher: You would also like to show your pictures?

Sara (overlapping): Me too. I can also show some.

Teacher: Great! Shall we do it now at once perhaps?

Elin: Yes (smiles)./—/

Sara: But I’ve got a whole movie.

Teacher: How long is it? Is it 40 minutes?

Sara: No/—/it’s only a couple of minutes long.

The girls got round the teacher with a humorous twinkle in their eyes to get time to show their pictures. The teacher realized that she would do better to start sharing the pictures so as to be able to do other things as well. What was symptomatic of these course meetings was the interplay between the girls and the teacher in which both parties seemed to accept walking the tightrope between keeping to the manual and upsetting it. The excerpt above demonstrates how a peripheral concluding exercise became a central, time-consuming one.

In the space the girls had been offered through DISA, between the ordinary school classes, they demonstrated in words and actions what was meaningful for them in order to feel well. Analysing the pictures they presented, and the stories they told during “sharing”, provided knowledge about the factors that acted on their psychological health. The main theme was affiliation and social relationships, especially those that last over time (Wickström, 2013). These long-lasting relations to family and friends seemed to constitute the biggest and most reliable support but included conflicts and trouble (Wickström & Zetterqvist Nelson, 2018). The fact that social relationships such as friends, families and social networks have a considerable impact on children’s and young people’s psychological health is confirmed in other studies of psychological health that stress children’s and young people’s perspectives (Armstrong, 2000; Johansson, Brunnberg, & Eriksson, 2007). However, the stories told and the questions raised during the classes demonstrated that the relationships were not easy to handle and not set once and for all. The girls’ stories revealed that they worked on their relationships in school and during leisure time. Each of them tried to build fellowship, create their space in a group and avoid isolation. Their efforts to be accepted, recognized and awarded status and popularity created solidarity as well as isolation (cf. Kostenius & Öhrling, 2006). They discussed the difficulties they had in socializing with each other; for example, one girl felt shut out by another girl who wanted to be left in peace for a while. The relationships constituted the ground for the girls’ psychological health and their stories showed how they tussled with each other, their family and their teachers and that their relationships created both positive and negative effects for their psychological health.

In different ways the girls showed the importance of their relations to friends, family and teachers for their well-being (cf. Wickström, 2013). The girls used the expression “friend sibling” to describe a good friend, whom the girls explained as “make-believe siblings” or “very good friends”. This term unites two of the most important aspects of children’s and young people’s well-being: friends and family. Throughout the course, the girls acted to shift the focus from individual cognitive manoeuvres to relational aspects of psychological health. To be able to create space for their urgent concerns, the girls were dependent on their teacher. She came to meet the girls and used her professional knowledge (beside her specific subject knowledge) to apply scientific methodology, act independently and suit her teaching to the specific group of students. Thus the teacher facilitated the girls’ actions. By getting space to weaken the rules in the manual and allowing themselves to speak up, the girls destabilized and decoded the educational assemblage (cf. DeLanda, 2006, p. 16). Their possibilities to act, however, were restricted to acting within the course. They did not find a possibility to meet in a smaller group together with the teacher outside the DISA programme. This demonstrates how the participants engaged with several forces and affected the assembling, but also to what extent the educational assemblage opened up possibilities.

### Cooperate and adopt

After being observant of the girls’ silences and upset, another thing that struck me during the observations was that the girls returned to the next meeting, despite the way they had reacted at the previous course meetings. What did they get out of a course that included many exercises in which they did not wish to take part? In fact, 63 of 85 DISA exercises concern registering negative or irrational thoughts and trying to handle them. Nevertheless, the girls cooperated and continued to come; partly it was a question of materiality and the institutional context. The course meetings seemed to represent an exclusive chance to meet in a small group with an interested and engaged teacher. In addition, the school was cramped for rooms and the DISA course was prioritized when it came to reservations. The educational assemblage opened up possibilities for being together and provided a room for a small group of schoolchildren. Thus the girls attempted to keep the activity in place. They were positive about exercises that presented a constructed story, often in the form of a comic strip. These tasks attracted the girls’ interest more than open questions about negative thoughts. It was not that the girls agreed with the message in the stories, but these examples of *concrete situations* triggered them to discuss and ask questions.

The next excerpt illustrates a situation where the teacher started an exercise entitled “Three ways to handle stressful situations”. She showed three sheets of papers with the headings:

1. Avoid the situation; 2. Change the situation; and 3. Change the way you react to the situation:

Teacher (reads from the manual): “You find yourself having lots of disheartening thoughts about yourself when you are with Johanna, who you think is perfect: good-looking, popular and the boys always flock around her.”

(Sara rocks on her chair and Pia yawns.)

Teacher: Let’s do like this (spreads the three sheets in front of the girls) so you can stand on one, so you can move.

(Sara remains seated but stretches out a foot on the sheet “Avoid the situation”.)

Teacher: Yes, ok, what do *you* think Pia?

(Pia walks over to the sheet “Change the situation”.)

Teacher (to Sara): Aha, ok, how could you avoid that situation?

Sara (thinks): Well, it’s just to think positive, perhaps I’m not more good-looking than her, but I am more good-looking than everybody else.

The message in this exercise is that disheartening thoughts can develop when you are with someone else who is good-looking and popular. Longing for better looks and more popularity is ascribed to the participants (cf. Wickström, 2013). This episode demonstrates that, in spite of the girls’ resistance to speaking about negative thoughts, Sara has adopted the course logic of changing negative thoughts. But the excerpt also shows that the idea of CBT, to change an incorporated behaviour to think negatively about yourself and the surroundings (Beck, 1979), has been through a looping effect and replaced by something more akin to simplified and popular positive thinking as an ideology where negative emotions should be repressed. In cooperating and adopting the logic of the course, interactive processes develop between concepts of psychological health and the girls who use them (cf. Hacking, 1999).

In another exercise the girls are shown comic strips in which they have to identify the negative thought and depressive feeling that is presented. They are further asked to create a positive counter-thought and present the better feeling coming out of it. In the following excerpt the teacher shares a comic strip in which a girl, on the left in the picture, talks about “super beautiful girls” who are “bitches” and explains how much she likes her friend who is “kind and very average”:

Teacher: What negative thoughts do you think the girl on the right has? “I want to be like them, she does not think I am pretty ….” Who wants to be average? You want to be pretty … perhaps? What could be a positive counter-thought?

(The girls do not answer.)

Teacher: Now you have to work, you can fill in your answers.

(The girls start to write down their suggestions.)

Elin: But this depressive feeling, what can I write there? Can I write like sad?

Teacher: Yes, emotion, low-spirited, sad.

The exercise and the teacher present stereotyped pictures, one of girls being beautiful and bitchy, and another of being kind, average and not beautiful. The girls resist answering but engage in writing positive counter-thoughts. Elin’s question illustrates the girls’ cooperation in the sense that they try to grasp the concepts used in the manual. She exemplifies “depressive feeling” with “sad”. Psychological ill health, and concepts used to describe its symptoms, seemed abstract to the girls, as has been demonstrated in earlier research as well (cf. Johansson et al., 2007). The depressive symptom referred to in the exercise went through a looping effect and was labelled as sad, which is a normal emotional reaction. Through engaging in the exercise, however, the girls adopted the practice of working with cognitive techniques. When they cooperated and elaborated on the concepts, they accepted the rules for the course and got involved in coding it. To some extent they adopted the logic of the course (cf. Bollig & Kelle, 2016), which restabilized the educational assemblage.

## Discussion

### Individual problems and gendered expectations

Public health initiatives since the end of the 1990s, as described in the introduction, have mainly been directed at whole populations in order to prevent psychological ill health from occurring. Psycho-educational programmes, such as DISA, represent a material reality of public health efforts governed by biomedical diagnostic systems and implemented in schools. In this article, I study how a DISA course works as an educational assemblage that starts processes of territorialization. I demonstrate that 13-year-old schoolgirls who participate in a psycho-educational programme became part of an assemblage that comprised:

manual—gender—teacher—peers—classroom—CBT—individual thoughts

The relations the girls had with the manual and its content, the CBT techniques, the teacher and peers, affected the girls in that they took part in 13 course meetings. The girls worked to a great extent with the exercises in the manual and they adopted concepts such as depressive thoughts and thinking positively. The DISA programme consists of elements that come from outside, presenting concepts of psychological health and techniques to improve young girls’ psychological health. The programme situates psychological ill health within individual children and asks the participants to take responsibility for any problems they are having. The girls were “made up” (Hacking, 1995) to think about themselves in relation to the exercises and the concepts used.

The educational assemblage created normative gender positions and asked exclusively girls to achieve on a psychological level: they should avoid negative thoughts (cf. Gunnarsson, 2015). The girls were presented with a large number of pictures and ideas about their bodies, clothes and appearances, ideas that the teacher established when trying to involve the girls. An identity was built for the participants made up of stereotyped thoughts about external form (clothes and appearances), heteronormative relations (boys flocking around a good-looking girl), homosocial relations (girls are with girls, not with boys) and social approval (being popular). The girls were thought to have problems with negative thoughts about their bodies and appearances (cf. Kvist Lindholm & Zetterqvist Nelson, 2014; Wickström, 2013); at the same time they were saddled with the responsibility not to let these aspects bother them. The girls kept silent and seemed to reject the positions created for them. The oral evaluation the teacher initiated demonstrated that they found DISA discriminating in that it primarily targeted girls. The course put expectations on them to have problems with their thinking, while boys in the same school found themselves excluded from discussing psychological health issues. The exclusion of a certain category is a territorialization which increases the internal homogeneity of the educational assemblage (cf. DeLanda, 2006, p. 13). The assemblage connected the girls to norms about girls’ specific roles and obligations, and to logic about psychological health as located in their minds, without admitting the impact of external forces.

### Health agency: altering the condition for the assemblage

Studying agency, it is tempting to look for the rebellious and the energetic. Educational programmes, however, create a framework that is difficult for teachers as well as schoolchildren to defend themselves against. Therefore, it is important to look for both the possibilities and the restrictions an educational assemblage offers. While normative positions were built for girls in this study, they also acted upon and made use of the programme by being *silent*, *upset* and *cooperative* on the course. They adhered to the course but refrained from taking part in some of the exercises such as the extensive work on identifying negative thoughts. The assemblage opened up possibilities for the girls in that the teacher was flexible and willing to adjust the exercises to this specific group of students. The analysis of the girls’ health agency, their acts and stories, seen in their constant efforts to bring in their urgent concerns, demonstrated that psychological health was not experienced as an individual psychological issue but as a complex relational and social matter. Children’s and young people’s relationships are in constant flux and they work on them on a daily basis at home and in school. The participants’ identification of problems, as well as the solutions to them, did not fit the templates used in the programme. In fact, the assemblage was elaborated into:

manual—gender—teacher—peers—classroom—interaction—relations

The theoretical foundation for the intervention, drawn from CBT and clinical practice for people diagnosed with depression, was not suited to deal with complicated interactional questions of young people’s everyday lives, nor with their context-sensitive well-being. The elaboration the girls contributed raises critical questions about the course logic—to prevent psychological ill health by letting students, no matter how they are, devote themselves to interpreting and working on their thoughts and emotions. The concepts used went through a looping effect. On the one hand, the psychiatric concepts seem to lose their meaning and become commonplace and general, with the possible effect that those who need help in a psychological sense run the risk of not being attended to. On the other hand, when emotional expressions are defined by concepts used in the treatment of depressed people, there is a risk that everyday experiences of being nervous, worried or sad are formulated as divergent and as signs of psychological ill health instead of normal reactions to being human and as existential dimensions of life.

Exploring the participating girls as health actors casts light on their practices. The results demonstrate that they took action and altered the assemblage. Their practices reveal that the rules governing the assemblage are based on the assumption that the solution to young people’s psychological problems is to teach them skills to change their thoughts about the world, not work on the world itself. Research studies on the realization of DISA and other manual-based psycho-educational programmes show that these programmes disregard children’s and young people’s own identification of problems as well as their understanding of what is significant for their psychological health and well-being (Kvist Lindholm, 2015; Morrow & Mayall, 2009; Wickström, 2013). Taking young people’s perspectives into consideration reveals how the girls experience their psychological health. First of all, they resist the ready-made problem focus that locates psychological health in their thinking; instead, they direct the focus on the relational aspects that they see as the basis for their psychological health. Secondly, they minimize the time they spend on exercises based on distant and general assumptions about negative thoughts being a problem; instead, they try to devote their time to their actual and concrete assets and problems. Their choice of exercises is a critique of universal manual-based programmes developed from CBT treatment.

The logic of the course was nevertheless adopted in that the girls were “made up” (Hacking, 1995) to think about themselves in relation to, for example, the concepts of “positive thoughts” and “depressive states”. These instances of adhesion also demonstrate that the logic of the course went through a looping effect (Hacking, 1995): the meaning of the concepts changed to “being positive” and “being sad”, which impoverished the original meaning and turned normal life conditions into symptoms of psychological ill health. Thus the DISA assemblage was further elaborated into:

manual—gender—teacher—peers—classroom—interaction—relations—positive thinking—pathology

Other possibilities were restricted for the girls, such as having an impact on how the school planned activities to promote their psychological health. The girls were left with elaborating the DISA assemblage. Their agency was an effect of the practice they took part in (cf. Bollig & Kelle, 2016). The schoolgirls acted and destabilized the educational assemblage, but there were no radical changes of it outside these specific classes (cf. Youdell & McGimpsey, 2015). However, their acts of upsetting and making the course meaningful constitute knowledge about the factors that affect their psychological health.

In this research, assemblage ethnography has been the ground for an investigation of how heterogeneous actors interact (human agents—for example, a teacher—as well as non-human agents, in this case the DISA manual and CBT exercises); how they build agency for a group of schoolgirls, and how the girls use their possibilities to act. Children depend on multiple factors for their agency; the institutional networks they are involved in both allow and restrict their actions. The study demonstrates that focusing on children as health actors, in the sense that agency develops in the assemblages children take part in and in relation to other agents in these assemblages, can complement the knowledge base and question the framing of psychological health. The relational, gendered and variable aspects of psychological health that the girls’ actions prove constitute the cultural and social context that cannot be ignored if we are to understand and approach children’s and young people’s psychological health.

## Transcription conventions

(text) indicates transcriber’s comments

… pause

**bold** emphasis

/—/talk omitted from the data excerpt

“text” dialogue within the story
